# Reduced Functional Connectivity within the Mesocorticolimbic System in Substance Use Disorders: An fMRI Study of Puerto Rican Young Adults

**DOI:** 10.3389/fnbeh.2016.00102

**Published:** 2016-05-25

**Authors:** Jonathan Posner, Leora Amira, Antonio Algaze, Glorisa Canino, Cristiane S. Duarte

**Affiliations:** ^1^Department of Psychiatry, Columbia College of Physicians and Surgeons, Columbia UniversityNew York, NY, USA; ^2^Department of Psychiatry, New York State Psychiatric InstituteNew York, NY, USA; ^3^Department of Radiology, University of Puerto RicoSan Juan, Puerto Rico; ^4^Behavioral Sciences Research Institute, Academic Affairs, School of Medicine, University of Puerto RicoSan Juan, Puerto Rico

**Keywords:** functional MRI, mesocorticolimbic system, connectivity, substance use disorder, nucleus accumbens, impulsivity

## Abstract

Studies of the mesocorticolimbic reward system (MCLS) and its relationship with impulsivity and substance use disorders (SUD) have largely focused on individuals from non-minority backgrounds. This represents a significant gap in the literature particularly for minority populations who are disproportionately affected by the consequences of SUD. Using resting-state functional MRI (fMRI), we examined the coherence of neural activity, or functional connectivity, within the brain’s MCLS in 28 young adult Puerto Ricans (ages 25–27) who were part of a population-based cohort study. Half of the sample lived in San Juan, Puerto Rico; the other half lived in the South Bronx, New York. At each of the two sites, half of the sample had a history of a SUD. Relative to those without SUD, individuals with SUD had decreased connectivity between the nucleus accumbens (NAcc) and several regions within the MCLS. This finding was true irrespective of study site (i.e., San Juan or South Bronx). Reduced connectivity within the MCLS was also associated with higher self-reported levels of impulsivity. Path analysis suggested a potential mechanism linking impulsivity, the MCLS, and SUD: impulsivity, potentially by chronically promoting reward seeking behaviors, may contribute to decreased MCLS connectivity, which in turn, may confer vulnerability for SUD. Expanding upon prior studies suggesting that alterations within the MCLS underlie SUD, our findings suggest that such alterations are also related to impulsivity and are present in a high-risk young minority population.

## Introduction

MRI studies suggest that substance use disorders (SUD) and impulsivity are both associated with atypical neural connections within the brain’s mesocorticolimbic reward system (MCLS; Gu et al., 2010; Tomasi et al., 2010; Upadhyay et al., 2010), which includes the midbrain’s ventral tegmental area (VTA), the nucleus accumbens (NAcc), and the orbitofrontal cortex (OFC). However, the inter-relationships between the MCLS, impulsivity, and SUD remain poorly characterized.

Substance use is associated with increased extracellular dopamine (DA) within the MCLS (Volkow et al., 2007). Increased dopaminergic outflow from the VTA to the NAcc is thought to encode stimuli associated with novel and unexpected outcomes, such as illicit drugs and their related euphoric effects. Encoding stimuli (e.g., a drug of abuse) with a reinforcer (e.g., the euphoric response to the drug), at least in part, underlies reinforcement learning and motivates behaviors to obtain more of the desired outcome. Because of the relationship between the MCLS and reinforcement learning, numerous neuroimaging studies have examined the MCLS in individuals with SUD.

Despite the many advances made by prior neuroimaging studies of the MCLS, important questions persist. Altered connectivity of the MCLS has been associated both with SUD and impulsivity, and behavioral research similarly indicates an association between SUD and impulsivity (Volkow et al., 1999; Lejuez et al., 2002; Posner et al., 2009, 2014b). However, the inter-relationships across these three variables need to be better understood. As suggested elsewhere, by chronically promoting reward-seeking behavior, impulsivity may alter the MCLS and these alterations in the MCLS may, in turn, confer risk for SUD (Posner et al., 2013; Plichta and Scheres, 2014). However, to develop a clearer mechanistic understanding, further clarity is still needed on how impulsivity, SUD, and MCLS connectivity are inter-related.

Also of relevance, knowledge about connectivity within the MCLS and its relationship with SUD are based on studies focused on non-minority, highly selected populations in the US (Pierce and Kumaresan, 2006; Gu et al., 2010; Tomasi et al., 2010; Upadhyay et al., 2010). MCLS connectivity and its relationship with SUD has never been examined in a minority population. Given that the determinants (Clark et al., 2015) and consequences (Kakade et al., 2012) of SUD may differ depending on one’s racial/ethnic and socio-cultural background, the neural underpinnings of SUD could arguably differ across racial/ethnic groups. For example, processes underlying reinforcement learning and substance use may differ when the use of illegal substances can have dramatic consequences (e.g., arrest), as is more common among minorities relative to White youth (Kakade et al., 2012). Puerto Ricans, the second largest Latino subgroup in the US, have the highest rates of SUD among all US Latino subgroups (Alegria et al., 2006, 2007; Caetano et al., 2009). However, MCLS connectivity in Puerto Rican individuals with SUD has not been studied and it has scarcely been examined in Latinos in general.

We examined the MCLS and its relationship with SUD in individuals of Puerto Rican descent by studying participants from a large, epidemiological cohort study of Puerto Ricans living in the South Bronx, New York and the metropolitan area in San Juan, Puerto Rico. Toward this end, resting fMRI scans were obtained and analyzed from 28 young adult Puerto Ricans (ages 25–27). Half of the sample, living in San Juan metropolitan area, was scanned at the University of Puerto Rico (UPR). The other half of the sample, living in the Bronx, New York, was scanned at the New York State Psychiatric Institute (NYSPI). Both sites used the same GE 3-Tesla MRI platform (see below, “MRI Sequences” Section). At each site, half of the sample had a history of SUD. We hypothesized that relative to individuals without SUD (SUD−), those with SUD (SUD+) would have altered functional connectivity within the MCLS. Lastly, we conducted exploratory analyses to broaden our understanding of the relationship between MCLS connectivity, impulsivity, and SUD. Given the well-documented relationship between SUD and impulsivity (de Wit, 2009), we explored whether self-reported measures of impulsivity were associated with MCLS connectivity. Lastly, we used path analysis to explore a potential mechanism relating impulsivity, MCLS connectivity, and SUD.

## Materials and Methods

The Institutional Review Boards of the NYSPI and UPR approved the study procedures. All participants provided written informed consent.

### Participants

MRI data were obtained and analyzed from 28 young adults between the ages of 25 and 27 years (mean = 25.70 ± 0.67) who were recruited from a large, epidemiological study of Puerto Ricans in the South Bronx, New York and San Juan, Puerto Rico (Bird et al., 2006). Participants across the two sites were group-matched on age, sex, and presence of SUD and comorbid psychiatric disorders (Table 1). Diagnostic interviews, including assessments of SUD and comorbid psychiatric conditions, were conducted using the English or Spanish schedules of the World Health Organization (WHO) *Composite International Diagnostic Interview Version* (*CIDI*; Kessler and Ustün, 2004; Tobacco, Alcohol Use and Illegal SU (includes prescription drugs) modules). Clinical and demographic data are provided in Table 1.

**Table 1:** **Sample demographics**.

	SUD+ (*n* = 15)	SUD− (*n* = 13)	Test Statistic (df, *n*)	*p* value
Age	25.60 ± 0.63	25.77 ± 0.73	*t*_(26, 28)_ = 0.660	*p* = 0.515
Sex	10 M	7 M	$X_{\left(1,28\right)}^{2}$ = 0.480	*p* = 0.700
	5 F	6 F		
Scan site	8 NYSPI	7 NYSPI	$X_{\left(1,28\right)}^{2}$ = 0.001	*p* = 1.000
	7 Puerto	6 Puerto		
	Rico	Rico		
Types of SUD	1 AlD	N/A	N/A	N/A
	8 AlA
	4 DrD
	1 DrA
	1 AlA and DrD
Comorbidity	8 No	9 No	$X_{\left(1,28\right)}^{2}$ = 0.738	*p* = 0.460
	7 Yes	4 Yes		
Types of comorbid disorders	1 MDD	2 MDD	N/A	N/A
	3 BAD	1 GAD		
	1 GAD	1 PTSD		
	1 PTSD			
	1 BAD and PTSD
Education	14 HS or equivalent	13 HS or equivalent	$X_{\left(1,28\right)}^{2}$ = 0.912	*p* = 1.000
BIS total impulsivity	63.47 ± 8.84	60.92 ± 12.91	*t*_(26, 28)_ = −0.615	*p* = 0.544

*M, male; F, female; NYSPI, New York State Psychiatric Institute; AlD, alcohol dependence; AlA, alcohol abuse; DrD, drug dependence; DrA, drug abuse; MDD, Major depressive disorder; BAD, Bipolar affective disorder; GAD, Generalized anxiety disorder; PTSD, Post-traumatic stress disorder; HS, high school; BIS, Barratt impulsivity scale; N/A, not applicable*.

### MRI Sequences

Images were acquired at NYSPI and UPR. Both sites used the same MRI platform: GE MR750 3.0 Tesla whole body scanner. T1-weighed sagittal localizing images were acquired, followed by a high-resolution anatomical image for coregistration with axial echoplanar images. Axial echoplanar images (TR = 2200 ms, TE = 30 ms, 90° flip angle, receiver bandwidth = 62.5 kHz, single excitation per image, slice thickness = 3.5 mm, no gaps, 24 × 24 cm field of view, 64 × 64 matrix) were obtained to provide an effective resolution of 3.75 × 3.75 × 3.5 mm and whole-brain coverage. For resting-state image acquisition, participants were instructed to remain still with their eyes closed and to let their minds wander freely. Two 5-min resting-state scans were obtained for each participant.

### Image Processing

Standard image preprocessing methods were used, employing SPM8 Software^1^ with the conn_toolbox^2^ for functional connectivity analysis (Posner et al., 2013, 2014c). The functional images were slice time and motion corrected, coregistered with a high-resolution anatomical scan, normalized into the Montreal Neurological Institute (MNI) space, resampled at 2 mm^3^, and smoothed with a Gaussian kernel of 6 mm^3^ full width at half maximum (FWHM; Friston et al., 1995). Connectivity preprocessing procedures followed the component-based noise correction method described elsewhere (Behzadi et al., 2007) to minimize non-neural influences on fMRI signal. Four participants were removed from the MRI analyses because of technical issues (e.g., excessive head motion, unable to tolerate MRI scan) leaving a sample of 28 participants for the MRI analyses.

Following preprocessing, the resting-state fMRI time series data were correlated voxel by voxel for each participant across the complete resting-time series. Fisher z transformation was applied. We generated connectivity maps for each subject with the seed region in the nucleus accumbens (NAcc) bilaterally. For the NAcc seeds, we created bilateral spherical masks (radius = 4 mm) centered on the stereotactic coordinates (MNI coordinates: Left NAcc: x, y, z = −9, 9, −8; Right NAcc: x, y, z = 9, 9, −8) as delineated in prior resting state-functional connectivity MRI (rs-fcMRI) studies (Di Martino et al., 2008; Posner et al., 2014a). We chose the NAcc as the seed region because it is a central node within the MCLS, and because prior research indicates that this seed is effective in assessing MCLS connectivity (Posner et al., 2013; Cha et al., 2015).

### Hypothesis Testing

To test our hypothesis that relative to SUD− individuals, SUD+ individuals would have altered functional connectivity within the MCLS, we entered the seed-based connectivity maps into second-level, random-effects general linear models (GLM) with Group as the single factor with two levels (SUD+ and SUD−), and comorbid disorders as a covariate (dummy coded 1 or 0). Given our *a priori* hypothesis regarding MCLS connectivity, we anatomically restricted these group comparisons to the following MCLS regions: OFC, amygdala, hippocampus, NAcc, and midbrain/VTA. Masks of these regions were derived from the WFU_pickatlas (Maldjian et al., 2003). Exploratory whole-brain analyses were also conducted (see Supplementary Material).

To test whether the relationship between SUD and altered MCLS connectivity differed at the two sites, we conducted a moderator analysis. The dependent variable was MCLS connectivity, and the independent variables were Group (SUD+ and SUD−), Site (South Bronx and San Juan), and a Group by Site interaction term.

### Exploratory Analyses

All exploratory analyses were limited to MCLS regions in which significant differences in functional connectivity were detected between SUD+ and SUD− participants during hypothesis testing.

### Relationship with Impulsivity

We examined associations between a self-report measure of impulsivity (Barratt Impulsiveness Scale, BIS-11(Barratt, 1994)) and MCLS connectivity. For regions in which MCLS connectivity differed between SUD+ and SUD− participants, we calculated partial correlations between: (i) MCLS connectivity and (ii) impulsivity, while controlling for site and group (SUD+ and SUD−).

### Path Analysis

We used path analysis (Hayes, 2013) to further explore associations between MCLS connectivity, impulsivity, and SUD. Although path analyses can imply causal relationship between the variables of interest, our cross-sectional study cannot impute causality. Thus these analyses and results should only be interpreted as supportive, or preliminary, in nature.

### Correction for Multiple Statistical Comparisons

We corrected for multiple statistical comparisons. We used the small volume correction function within SPM with the following regions derived from WFU_pickatlas (Maldjian et al., 2003): OFC, amygdala, parahippocampal gyrus, and midbrain. Clusters with a family wise error corrected alpha of <0.05 (*p*_fwe_ < 0.05) were considered statistically significant.

### Head Motion during fMRI Scanning

As recommended elsewhere (Power et al., 2011), we examined the potential confounding influence of head motion to our resting state fMRI data by calculating root mean square (RMS) and peak/average (across volumes) Framewise Displacement (FD). These calculations were based on each individual’s six head alignment parameters generated by SPM during its realignment procedure. We differentiated the six head realignment parameters across frames, and then calculated instantaneous head motion as a scalar in each frame, using the following formula: FDi = |Δd_ix_| + |Δd_iy_| + |Δd_iz_| + |Δα_i_| + |Δβ_i_| + |Δγ_i_|, where Δd_ix_ = d(_i − 1)x_ - d_ix_, and similarly for the other rigid body parameters [d_ix_ d_iy_ d_iz_ α_i_ β_i_ γ_i_]. We converted rotational displacements from degrees to millimeters by calculating displacement on the surface of a sphere of radius 50 mm. Group differences in the head motion parameters were tested using the non-parametric Mann-Whitney *U* test. There were no group differences in any of the head motion parameters (*p*’s > 0.2; Supplementary Figure 1) and head motion was minimal in both groups: RMS in SUD+ group = 0.44 ± 0.3 mm; RMS in SUD− group = 0.42 ± 0.5 mm; mean peak FD in SUD+ group = 0.29 ± 0.2 mm; mean peak FD in SUD− group = 0.23 ± 0.4 mm). Motion parameters were included as nuisance regressors both at the subject- and group-level.

## Results

### Hypothesis Testing

**Figure 1:**
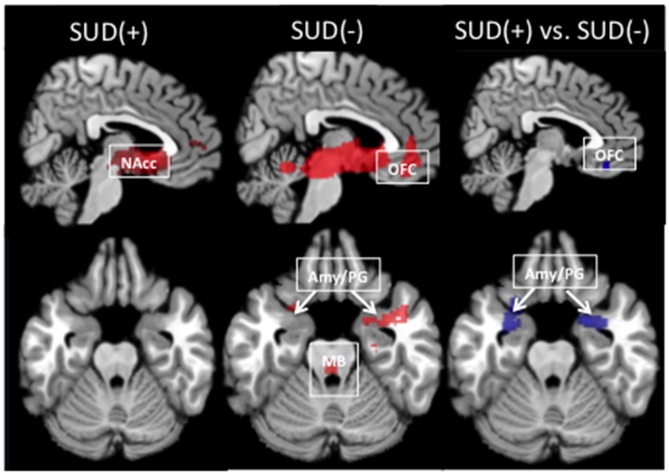
**Resting-state functional connectivity maps with the seed in the left nucleus accumbens (NAcc).** In individuals with and without a substance use disorder (SUD+ and SUD−), the NAcc seed was positively correlated with regions within the mesocorticolimbic system, including the medial orbitofrontal cortex (OFC), amygdala and parahippocampal gyrus (Amy/PG), and midbrain/ventral tegmental area (MB). Relative to SUD− participants, SUD+ participants showed decreased connectivity between the NAcc and the OFC and Amy/PG (*p*_fwe_ < 0.05). Red indicates positive connectivity. Blue indicates connectivity that is reduced in SUD+ relative to SUD−.

In participants with and without SUD, the seed-based connectivity maps generated from the left and right NAcc showed connectivity with regions within the MCLS including the medial OFC, midbrain/VTA, parahippocampal gyrus, and amygdala (Figure 1).

#### Left NAcc

Relative to SUD− participants, SUD+ participants showed significantly reduced connectivity of the left NAcc with the left OFC, left amygdala, and left parahippocampal gyrus (Figure 1; Table 2). SUD+ participants also showed reduced connectivity between the left NAcc and the right parahippocampal gyrus, but this effect was subthreshold (*p*_fwe_ = 0.08). There were no regions in which SUD+ participants showed increased MCLS connectivity relative to SUD− participants.

**Table 2:** **MCLS connectivity**.

		MNI coordinates		
Seed Region		*x*	*y*	*z*	Hemisphere	Cluster size (voxels)	peak *t*	*p*_fwe_
Left nucleus accumbens	**SUD(−) > SUD(+)**
	Amygdala	−28	0	−28	L	276	4.49	0.003
	Orbitofrontal cortex	−2	38	−20	L	75	3.69	0.04
	Parahippocampal gyrus	−24	−34	−16	L	226	4.27	0.02
	Parahippocampal gyrus	30	−36	−6	R	155	3.67	0.08*
	**SUD(+) > SUD(−)**
	N/A
Right nucleus accumbens	**SUD(−) > SUD(+)**
	Parahippocampal gyrus	−20	−42	−2	L	331	5.04	0.003
	Parahippocampal gyrus	24	−36	−8	R	133	3.97	0.04
	Midbrain/VTA	−4	−26	−8	R/L	154	3.48	0.1*
	Amygdala	−30	0	−26	L	323	5.21	0.1*
	**SUD(+) > SUD(−)**
	N/A

MCLS, mesocorticolimbic system. L, left; R, right; R/L bilateral; N/A, not applicable; MNI, Montreal Neurological Institute. *Did not meet statistical threshold of p_fwe_ < 0.05.

#### Right NAcc

Relative to SUD− participants, SUD+ participants showed reduced connectivity of the right NAcc with left and right parahippocampal gyrus (Table 2). SUD+ participants also showed reduced connectivity, though subthreshold (*p*’s_fwe_ = 0.1), between the right NAcc and the left amygdala, as well as between the right NAcc and the midbrain/VTA. There were no regions in which SUD+ participants showed increased MCLS connectivity relative to SUD− participants.

#### Effects of Site

For regions identified during hypothesis testing as differing between SUD+ and SUD− participants, we examined potential effects of site (i.e., South Bronx and San Juan). Neither a main effect of site, nor the group (SUD+ vs. SUD−) by site interaction was significant (*p* > 0.3). For each site, bar graphs and statistical tests are provided in the Supplemental Materials.

#### Exploratory Analyses

All exploratory analyses were restricted to regions identified during hypothesis testing as differing significantly between SUD+ and SUD− participants.

#### Relationship with Impulsivity

**Figure 2:**
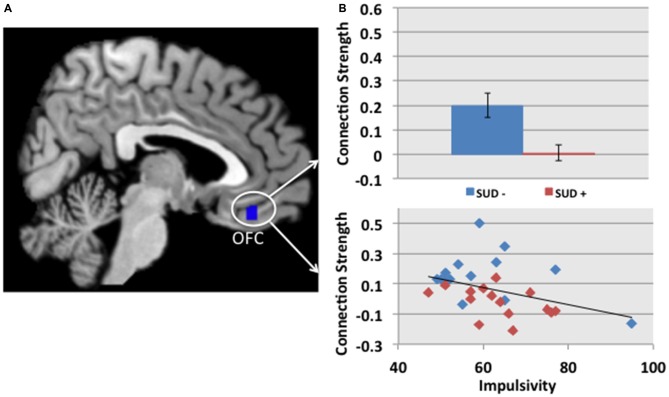
**(A)** The cluster in blue depicts the region within the OFC, in which connectivity with the left NAcc is reduced in participants with a SUD+ vs. SUD−. **(B)** The connection strength between the left NAcc and the OFC correlated with impulsivity, as measured by the Barratt Impulsiveness Scale, BIS-11 (Barratt, 1994; *r* = −0.45 *p* = 0.02). This partial correlation controlled for site and group (SUD+ or SUD−). In the scatterplot, blue and red diamonds represent SUD− and SUD+ participants, respectively.

While controlling for site and group (SUD+ or SUD−), we found that impulsivity correlated inversely with the connection strength between the left NAcc and the left OFC (*r* = −0.45 *p* = 0.02, Figure 2). The finding was significant based on Pearson and Spearman rank correlations, and remained significant after excluding a participant with an impulsivity score of 95 (2 SD greater than the group mean). We did not find significant associations between impulsivity and the other MCLS regions for which connectivity differed between SUD+ and SUD− participants.

#### Path Analysis

**Figure 3:**
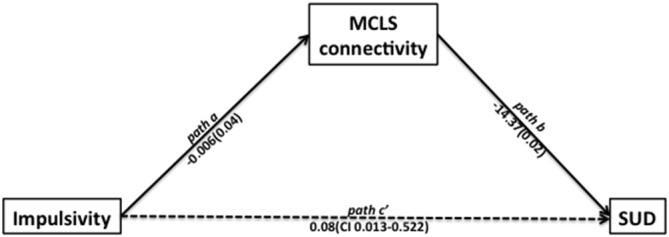
**Path analysis indicated that impulsivity is related to the likelihood of participants having a SUD and to the mesocorticolimbic system (MCLS) connectivity.** In the first linear regression model, we found that impulsivity was significantly associated with decreased MCLS connectivity (beta = −0.006, *t* = −2.13, *p* = 0.043). In the second linear regression model, we found that while controlling for impulsivity, MCLS connectivity was significantly associated with SUD (beta = −14.37, *z* = −2.27, *p* = 0.024, Wald = 5.13). Lastly, we used bias corrected bootstrapping to confirm the significance of the indirect effect of impulsivity on SUD in the presence of decreased MCLS connectivity (coefficient = 0.081; 95% CI = 0.013–0.522).

Using path analysis (Hayes, 2013), we found support for a potential mechanism relating impulsivity, MCLS connectivity, and SUD. This analysis was consistent with a model suggesting that impulsivity may contribute to the development of SUD through its effects on the MCLS (Posner et al., 2013; Plichta and Scheres, 2014). In the first linear regression model, we found that impulsivity was significantly associated with low MCLS connectivity (left NAcc − left OFC connection strength; beta = −0.006, *t* = −2.13, *p* = 0.043). In the second linear regression model, we found that while controlling for impulsivity, MCLS connectivity was a significant predictor of SUD (−14.37, *z* = −2.27, *p* = 0.024, Wald = 5.13). Lastly, we used bias corrected bootstrapping (Hayes, 2013) to confirm the significance of the indirect effect of impulsivity on SUD as mediated by MCLS connectivity (coefficient = 0.081; 95% CI = 0.013–0.522, Figure 3).

## Discussion

This is the first MRI study to examine neural correlates of SUD within a population-based sample of Puerto Rican young adults (South Bronx, NY and San Juan, Puerto Rico). First, we found that relative to those without SUD, individuals with SUD have decreased connectivity between the NAcc and several regions within the MCLS. Second, we found that reduced connectivity between specific MCLS regions (the left NAcc and the left OFC) was associated with higher levels of impulsivity. Lastly, path analysis offered initial support for a mechanistic account of these findings: impulsivity may contribute to decreased MCLS connectivity, which in turn, may confer vulnerability for developing SUD.

Animal studies using microdialysis techniques indicate that addictive drugs increase extracellular concentrations of DA in the NAcc (Hurd et al., 1989; Pontieri et al., 1995). Human neuroimaging similarly suggests that addictive drugs, such as cocaine, increase extracellular DA within the MCLS, as indexed by decreases in striatal [^11^C]raclopride binding (Volkow et al., 2009). Cocaine-induced increases in striatal DA correlate with subjective reports of the euphoric effects of the drug (Volkow et al., 2004). Over time, persistent drug abuse disrupts the MCLS DA pathways. For example, individuals with SUD show attenuated DA responses to rewarding stimuli that are not drug-related, and a concomitant reduction in MCLS DA receptors (Volkow et al., 2004). Consistent with reports of attenuated MCLS DA responses in individuals with SUD, we found that individuals with SUD have reduced functional connectivity within the MCLS. Similar findings of reduced MCLS connectivity in SUD have been reported elsewhere (Gu et al., 2010; Tomasi et al., 2010; Upadhyay et al., 2010; Wang et al., 2010), although increased MCLS connectivity has also been reported (Ma et al., 2010; Wilcox et al., 2011). Youth at high risk for SUD based on family history may also have disrupted MCLS connectivity with some research suggesting decreased connectivity (Cservenka et al., 2014), and others suggesting increased connectivity (Weiland et al., 2013). Differences in findings (increased vs. decreased connectivity) could reflect heterogeneous effects of chronic vs. acute drug intake, differences in substances of abuse, and/or MRI methodologies (e.g., seed-based vs. independent component analysis; Sutherland et al., 2012). These disparate findings notwithstanding, our data are consistent with prior studies suggesting that altered connectivity within MCLS may represent an important neural correlate of SUD.

Our findings add to the growing literature on MCLS connectivity and SUD in important ways. Ours is the first study to examine functional connectivity from a non-referred sample of Puerto Rican young adults. Consistent with prior reports from selected, non-minority populations, for whom the substance use experience may involve different reward and motivational processes, we found evidence of widespread reductions in MCLS connectivity in SUD+ individuals. MCLS connectivity may therefore represent a neural correlate of SUD that is generalizable to a high-risk minority population. In addition, we found that reduced MCLS connectivity in SUD participants was consistent irrespective of the site (i.e., South Bronx, NY or San Juan, Puerto Rico, USA). The consistency of the findings across the two study sites suggests that detecting altered MCLS connectivity in individuals with SUD is robust to differences in MRI sites, at least when both sites use the same MRI platform.

Our path analysis provides initial support for a mechanistic account of the relationship between SUD, MCLS connectivity, and impulsivity. Our findings suggest that impulsivity, possibly by chronically prompting behaviors aimed at obtaining short-term rewards, may contribute over time to reduced MCLS connectivity. Reduced MCLS connectivity, in turn, may increase the likelihood of developing SUD. A similar hypothesis relating the chronic effects of impulsivity to the functioning of the MCLS has been put forward with regards to ADHD (Plichta and Scheres, 2014), an impulse-control disorder that confers risk for the development of SUD. It is worth noting, however, that our data were cross-sectional in nature, and thus we cannot exclude hypotheses with alterative causal implications. In other words, our data are equally supportive of one model (Impulsivity leads to decreased MCSL connectivity and thereby contributes to SUD) as of a second model (SUD leads to decreased MCLS connectivity and thereby contributes to impulsivity). Although our cross-sectional data are agnostic regarding the relative validity of either model, we maintain that the first model is more likely based on theoretical grounds. Impulsivity is a trait that typically begins in childhood, predating the onset of SUD (de Wit, 2009). It is thus more parsimonious to hypothesize that impulsivity contributes to the development of SUD, than the reverse: SUD contributes to impulsivity. This theoretical rationale aside, subsequent research using a longitudinal design could more directly test these alternative hypotheses. Also, animal models could use techniques such as optogenetics to probe MCLS connectivity, while examining subsequent effects on impulsivity and the self-administration of addictive drugs.

Limitations of the study are important to consider. First, we stratified participants by SUD+ vs. SUD−, irrespective of different types of SUD. It is possible that different substances, when abused, confer unique connectivity disturbances; likewise, it is also possible that the quantity or frequency of substance use begets greater disruptions in MCLS connectivity. Subsequent research might test these hypotheses by comparing connectivity in individuals with different types of SUD and/or including a detailed assessment of the quantity and frequency of substance use. Second, several of the participants (*n* = 11) had a comorbid disorder. Though controlling for psychiatric comorbidities strengthened our hypothesis testing, we cannot entirely exclude potential residual confounding effects. The heterogeneity of our study sample in terms of comorbid disorders and types of SUD limits its specificity. However, the sample was selected from a large, population-based cohort. While this sampling strategy invariably introduces heterogeneity, it also promotes generalizability. Third, we used a subjective, self-report measure of impulsivity (BIS-11) and not an objective, neurocognitive measure. Although self-report measures may be subject to reporting biases, they nonetheless may provide a more robust assessment of trait-aspects of impulsivity than do state-dependent neurocognitive measures (Stanford et al., 2009). Fourth, our path analysis suggests that reduced MCLS connectivity may represent a mechanism by which increased impulsivity confers risk for SUD. However, we did not find a direct relationship between increased impulsivity and SUD (*p* > 0.5), though this relationship has been reported elsewhere (Perry and Carroll, 2008). It is possible that additional variables, such as access to illicit drugs, obscure a direct association between impulsivity and SUD, while a mediation effect is still detectable (Baron and Kenny, 1986). Fifth, the study involved two MRI sites. Although both sites used the same MRI scanner and pulse sequences, and comparable effects were seen at both sites, subtle differences in MRI parameters cannot be entirely excluded. Last, our sample was small, and as noted above, subtle effects of socio-cultural context on MCLS connectivity may have gone undetected.

In sum, our findings suggest that MCLS connectivity may represent an important neurobiological correlate of SUD not only among selected, non-minority populations, as demonstrated previously, but also in a high-risk, community-based, minority sample. Our findings also offer a potential heuristic elucidating the relationship between SUD, MCLS connectivity, and impulsivity. Although our study takes an initial step toward examining potential ethnic differences in relationship to the neurobiology of SUD, future studies with larger and potentially more diverse samples might continue this important line of inquiry.

## Author Contributions

JP, LA, and CSD: data collection, hypothesis testing, manuscript preparation; AA and GC: data collection, manuscript preparation.

## Conflict of Interest Statement

The authors declare that the research was conducted in the absence of any commercial or financial relationships that could be construed as a potential conflict of interest. JP has received research funding from Shire Pharmaceuticals.

## Footnotes

^1^http://www.fil.ion.ucl.ac.uk/spm/

^2^http://www.nitrc.org/projects/conn
