# Supplementary material for: Reduced Functional Connectivity within the Mesocorticolimbic System in Substance Use Disorders: An fMRI Study of Puerto Rican Young Adults
Source: Front Behav Neurosci. 2016 May 25;10:102. doi: 10.3389/fnbeh.2016.00102 (PMC4879128; doi:10.3389/fnbeh.2016.00102)
Supplement: Supplementary file 1 [file DataSheet_1.docx]

**Supplemental Material**

I. Whole Brain Connectivity Analyses

The whole-brain functional connectivity was analyzed using seeds in the left and right NAcc. We corrected for multiple statistical comparisons using a voxel-level statistical threshold (uncorrected p=0.005) and cluster filter (cluster size > 75 voxels). The results from the whole brain analysis are presented in Supplemental Table 1.

II. Effects of Socio-Cultural Context.

To test whether the relationship between SUD and altered MCLC connectivity differed by socio-cultural context, we split our sample into 2 sub-samples: those from South Bronx and those from San Juan. We repeated our GLM analysis testing the effect of Group (SUD+ and SUD-) on MCLS connectivity, separately for the South Bronx and San Juan sub-samples. All but one of the regions for which we detected reduced MCLS connectivity (based on left and right NAcc seeds) continued to show significantly reduced connectivity when the South Bronx and San Juan subsamples were analyzed separately (Supplemental Table 1 & Supplemental Figure 1).

**Supplemental Figure 1. Boxplots of head motion parameters of resting state fMRI of each group.** Numbers above the brackets denote significance of non-parametric Mann-Whitney U tests (2-tailed).


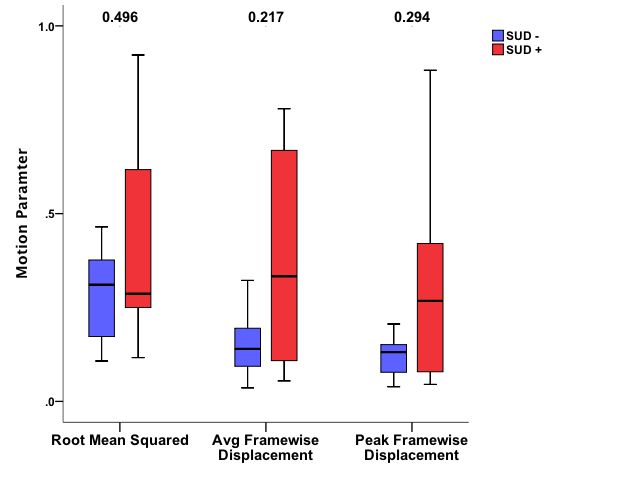

NAcc, Nucleus Accumbens, OFC, Orbitofrontal cortex, MCLS, Mesocorticolimbic system

| **Supplemental Table I**  **Seed Region** |  | **MNI Coordinates** | | |  |  |  |
| --- | --- | --- | --- | --- | --- | --- | --- |
| **Left Nucleus Accumbens** | **SUD(-) > SUD(+)** | **x** | **y** | **z** | **Hemisphere** | **cluster size (voxels)** | **peak t** |
|  | Amygdala | -28 | 0 | -28 | L | 276 | 4.49 |
|  | Orbitofrontal cortex | -2 | 38 | -20 | L | 75 | 3.69 |
|  | Parahippocampal gyrus | -24 | -34 | -16 | L | 226 | 4.27 |
|  | Parahippocampal gyrus | 30 | -36 | -6 | R | 155 | 3.67 |
|  | **SUD(+) > SUD(-)** |  |  |  |  |  |  |
|  | Anterior Prefrontal Cortex | 6 | 58 | -18 | R | 714 | 5.3 |
|  | Occipital Lobe | -18 | -86 | 2 | L | 1573 | 4.52 |
|  | Superior Temporal Gyrus | -68 | -30 | 18 | L | 96 | 5.27 |
|  | Cingulate Gyrus | 14 | -32 | 48 | R | 89 | 5.59 |
|  | Superior Frontal Gyrus | 6 | 0 | 72 | R | 141 | 3.29 |
|  |  |  |  |  |  |  |  |
| **Right Nucleus Accumbens** | **SUD(-) > SUD(+)** |  |  |  |  |  |  |
|  | Parahippocampal gyrus | -20 | -42 | -2 | L | 331 | 5.04 |
|  | Parahippocampal gyrus | 24 | -36 | -8 | R | 133 | 3.97 |
|  | Midbrain/ VTA | -4 | -26 | -8 | R/L | 154 | 3.48 |
|  | Amygdala | -30 | 0 | -26 | L | 323 | 5.21 |
|  | **SUD(+) > SUD(-)** |  |  |  |  |  |  |
|  | Anterior Prefrontal Cortex | 8 | 58 | -20 | R | 362 | 4.83 |
|  | Middle Frontal Gyrus | 36 | 56 | -14 | R | 83 | 3.73 |
|  | Anterior Prefrontal Cortex | -20 | 62 | -8 | L | 94 | 3.81 |
|  | Superior Temporal Gyrus | 60 | -12 | -2 | R | 107 | 3.76 |
|  | Superior Temporal Gyrus | -68 | -34 | 18 | L | 315 | 4.79 |
|  | Superior Frontal Gyrus | 14 | 20 | 68 | R | 156 | 3.8 |

**Supplemental Table II**

| **Left Nacc - Left Amygdala** | Mean connection strength | |  |  |
| --- | --- | --- | --- | --- |
|  | SUD+ | SUD- | t test | p value |
| South Bronx | 0.189 | 0.023 | 3.6 | 0.005 |
| San Juan | 0.153 | -0.053 | 3.4 | 0.006 |
|  |  |  |  |  |
| **Left Nacc - Left hippocampus** | Mean connection strength | |  |  |
|  | SUD+ | SUD- | t test | p value |
| South Bronx | 0.187 | 0.031 | 3.5 | 0.004 |
| San Juan | 0.163 | 0.019 | 2.7 | 0.02 |
|  |  |  |  |  |
| **Left Nacc - Left OFC** | Mean connection strength | |  |  |
|  | SUD+ | SUD- | t test | p value |
| South Bronx | 0.159 | 0.015 | 1.8 | 0.1 |
| San Juan | 0.245 | -0.006 | 2.9 | 0.02 |
|  |  |  |  |  |
| **Right Nacc - Left hippocampus** | Mean connection strength | |  |  |
|  | SUD+ | SUD- | t test | p value |
| South Bronx | 0.184 | 0.023 | 2.6 | 0.02 |
| San Juan | 0.238 | -0.023 | 4.7 | 0.001 |
|  |  |  |  |  |
| **Right Nacc - Right hippocampus** | Mean connection strength | |  |  |
|  | SUD+ | SUD- | t test | p value |
| South Bronx | 0.184 | 0.023 | 2.6 | 0.02 |
| San Juan | 0.238 | -0.023 | 2.9 | 0.01 |
